# Developing Functional Genomics Platforms for Fungi

**DOI:** 10.1128/mSystems.00730-21

**Published:** 2021-08-24

**Authors:** Lori B. Huberman

**Affiliations:** a Plant Pathology and Plant-Microbe Biology Section, School of Integrative Plant Science, Cornell University, Ithaca, New York, USA

**Keywords:** functional genomics, fungi, transcriptional profiling

## Abstract

Fungi are responsible for diseases that result in the deaths of over a million individuals each year and devastating crop infestations that threaten global food supplies. However, outside of a select few model organisms, the majority of fungal genes are uncharacterized. The roles of these genes in the biology of the organism, pathogenesis, and mediating interactions with the environment and other microbes are unknown. Historically, fungal gene characterization has primarily relied on classical genetic screens. However, advances in sequencing technology have enabled more rapid methods of gene functional characterization. Large-scale transcriptional profiling projects are one solution to generating hypotheses about fungal gene function. Together with other ‘omics techniques and newer tools that enable massively parallel mutant screens, knowledge of fungal gene function will be substantially improved. Understanding the function of fungal genes will be instrumental in increasing global food security, protecting ecosystems, and improving health outcomes.

## COMMENTARY

Fungi play a vital role in our ecosystem. Many fungal species are a key part of carbon cycling in the environment—degrading dead biomass and returning the nutrients to the soil. Some fungi associate with living plants, giving the plants access to nutrients, such as phosphate, from the environment (1). Other fungal species are part of the human and animal microbiome, where they may play a role in the development of the immune response (2).

**FIG 1:**
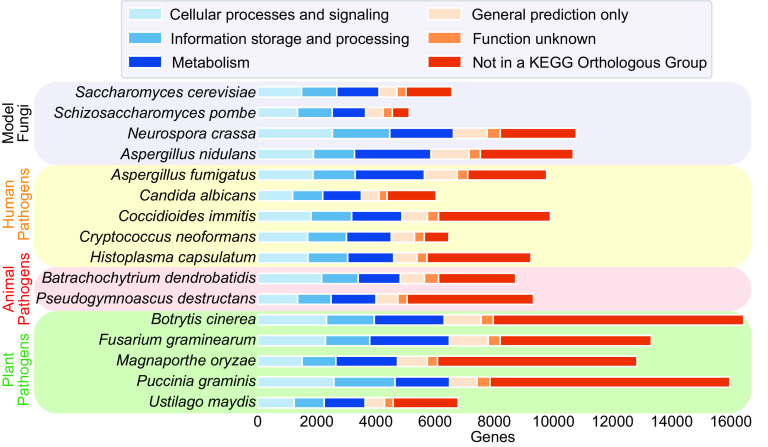
The function of many fungal genes is unknown. Graph of the number of genes in Kyoto Encyclopedia of Genes and Genomes (KEGG) Orthologous Groups in the indicated genomes. Gene categorizations into KEGG Orthologous Groups were based on annotations found on the Joint Genome Institute MycoCosm website (mycocosm.jgi.doe.gov).

Along with these beneficial roles, fungi infect both plants and animals. Fungal infestations of crops threaten global food security and are estimated to cause tens of billions of dollars per year in lost agricultural productivity (3, 4). Invasive fungal infections of humans are responsible for 1.6 million deaths per year (5). Among animals, fungi are causing catastrophic loss of bat and amphibian species (6, 7). Sadly, the impact of fungal disease is likely to increase as climate change allows fungal species to become endemic to new geographic locations or evolve to become more problematic pathogens (8). Despite all of this, outside of a select few model organisms, most fungal genes are uncharacterized or have only a very general functional prediction (Fig. 1). The roles of these genes in the biology of the organism, pathogenesis, and mediating interactions with the environment are unknown.

## STATUS QUO OF GENE CHARACTERIZATION

Historically, fungal gene characterization relied on classical genetic screens (9), a powerful tool used for decades to identify gene function (10, 11). Classical genetic screens create a direct connection from phenotype to genotype. This enables the generation of hypotheses about gene function that are tested using biochemical and cell biological assays. Unfortunately, genetic screens can be highly laborious outside of a select few model organisms. In obligate biotrophs and other unculturable fungal species, traditional genetic screens may be impossible.

Due to difficulties in performing genetic screens, it can be tempting to predict gene function using homology to characterized genes in well-studied fungi. However, while gains in gene characterization in model species such as Saccharomyces cerevisiae are enormous, extending that knowledge to other species has limitations. The roles of homologous proteins often diverge between species, especially when a gene family undergoes a large expansion within a particular lineage. An example of this is the family of zinc binuclear cluster transcription factors. The S. cerevisiae genome contains only 55 zinc binuclear cluster transcription factors, while the genome of the filamentous fungus Aspergillus nidulans contains 330 (12, 13). Such gene family expansions are common in fungi. Further, as the genomes of the most well-studied model fungi are relatively compact, many genes in fungal species of medical or agricultural interest have no characterized homologs (Fig. 1).

To meet the challenges fungi pose in medical and agricultural settings, it is important to characterize the functions of fungal genes. Advances in ‘omics technology create increasing numbers of tools for gene characterization. Transcriptional profiling provides key insights into fungal gene function and can be particularly powerful when used in combination with other ‘omics-based technologies. Going forward, the development of new tools will enable high-throughput functional genomics in fungi.

## USING SYSTEMS BIOLOGY TO IDENTIFY GENE FUNCTION

As next-generation sequencing technology is more readily available, large-scale RNA sequencing experiments are a more cost-effective mechanism for characterizing fungal genes. Transcriptional profiling of fungi—both those that can be cultured and unculturable fungi of the mycobiome—under a variety of conditions can help clarify gene and genome structure, particularly locations of exons and introns. This increases the accuracy of genome annotation and helps identify the relatively rare differential splicing events of fungal genes (14). Analyzing the sequences of these improved annotations helps place genes into families of homologs, improving functional predictions.

Along with improving gene annotation, transcriptional profiling elucidates gene expression levels. Fungal genes are often upregulated under conditions in which they play a role. Identifying upregulated genes helps generate hypotheses about gene function that can be tested using genetic, cell biological, and biochemical tools.

We used transcriptional profiling to identify the roles of several transcription factors associated with nutrient sensing in the filamentous fungus Neurospora crassa. In collaboration with the Joint Genome Institute, we transcriptionally profiled N. crassa during exposure to over 50 nutrient conditions. By identifying transcription factors that were upregulated under a specific condition, we made predictions about the roles of these transcription factors in nutrient sensing and utilization. To test these predictions, we used transcription factor deletion mutants to assay for changes in growth and gene expression patterns. This enabled the characterization of two transcription factors associated with carbon utilization (15).

Transcriptional profiling is useful for generating hypotheses about gene function, but it is strongest when used coordinately with other genetic tools. To better characterize the role the identified transcription factors played in nutrient sensing and utilization, we used DNA affinity purification sequencing (DAPseq). DAPseq is an *in vitro* method for identifying transcription factor DNA binding sites that distinguishes between direct and indirect transcription factor targets (16). DAPseq was instrumental in identifying the role of the transcription factor AMN-1, which is expressed most strongly when mannose is provided as the sole carbon source. AMN-1 regulates genes associated with mannose and mannan (a polysaccharide composed of mannose) utilization. However, DAPseq showed this regulation is indirect. A closer examination of the genes whose promoters AMN-1 does bind, suggested a role for AMN-1 in amino acid catabolism. Indeed, further experiments with an N. crassa mutant lacking *amn-1* showed that AMN-1 plays a key role in regulating amino acid utilization (17).

The use of DAPseq in combination with transcriptional profiling generated much more accurate hypotheses about the role of fungal transcription factors. However, DAPseq is not the only technology easily used in combination with RNA sequencing. Combining transcriptional profiling with other ‘omics technologies will be instrumental in assigning functions to uncharacterized genes. Along with indicating protein expression levels, proteomics provides information about patterns of protein modifications, which may help clarify the roles of genes involved in signaling pathways (18). As data sets of fungal metabolites improve, integrating metabolomics with transcriptional profiling will connect the role of fungal genes to the metabolic state of the cell (19). Going forward, the continuing development of new high-throughput methods of measuring biological activity will improve the power of systems biology in elucidating gene function.

## DEVELOPING NEW FUNCTIONAL GENOMICS TOOLS

The gold standard for generating hypotheses about gene function that can be tested using biochemical and cell biological tools is directly connecting phenotype to genotype. Historically, this is done using classical mutant screens in which a single mutant is analyzed at a time. Recently, however, several approaches for massively parallel mutant screens to get from phenotype to genotype in single celled organisms such as bacteria and some yeast species were developed. To do these massively parallel screens, pooled, barcoded mutant libraries are generated, typically by some form of insertional mutagenesis. Each barcode is associated with its corresponding mutated gene using high-throughput sequencing. The pooled mutant library is then exposed to different experimental conditions. Quantitative sequencing of the DNA barcodes determines the relative abundance of each mutant in the population. By identifying conditions in which a mutant is over- or underrepresented compared to the population as a whole, it is possible to make hypotheses about the function of the mutated gene.

Pooled mutant screens have changed the landscape of bacterial genetics, associating thousands of genes of unknown function with phenotypes (20). Massively parallel mutant screens have also been used in some yeast species. Pooled mutant screens recently enabled the identification of genes involved in lipid and alternative carbon metabolism in the basidiomycete yeast Rhodosporidium toruloides (21, 22). A much smaller and more targeted library was used to identify virulence factors in the corn smut pathogen Ustilago maydis (23). We are currently working to expand massively parallel mutant screens to filamentous fungi where we must develop tools to overcome the challenges of multinucleate cells, asexual cell fusion, and low transformation rates.

## EXPANDING TOOL SETS

Techniques like high-throughput functional genomics, transcriptional profiling, and DAPseq show promise in reducing the deficit in fungal gene characterization. These and other ‘omics tools used to identify the function of fungal genes will help us better understand how fungi interact with their environment and identify targets to treat fungal diseases of plants, animals, and fungi.

Although the examples discussed here involve fungi that can be genetically manipulated, techniques such as transcriptional profiling combined with *in vitro* tools like DAPseq could better our understanding of gene function in fungi that are currently unculturable. Additionally, improving the breadth of fungal gene characterization among culturable species across the fungal evolutionary tree will enhance the utility of using homology to predict gene function in related, unculturable fungal species. Understanding more about fungal pathogenesis and the role of fungi in the environment will help increase global food security, protect our ecosystems, and improve health outcomes.
